# Injectable gelatin microspheres loaded with platelet rich plasma improve wound healing by regulating early inflammation

**DOI:** 10.7150/ijms.51060

**Published:** 2021-03-03

**Authors:** Shaolong Zhou, Li Li, Chen Chen, Yi Chen, Linhua Zhou, Fiona H. Zhou, Jianghui Dong, Liping Wang

**Affiliations:** 1 Aesthetic Medical School, Yichun University, Yichun, 336000, Jiangxi, China.; 2 UniSA Clinical & Health Sciences, University of South Australia, Adelaide, SA 5001, Australia.; 3 School of Medicine, University of Adelaide, Adelaide, South Australia, 5000, Australia.

**Keywords:** platelet-rich plasma, gelatin microspheres, early inflammation, wound healing, tissue regeneration

## Abstract

We investigated the potential of gelatin microspheres (GMs) loaded with platelet-rich plasma (PRP) to enhance their wound healing effect. Platelets from the PRP were immobilized onto GMs to form biomimetic bioreactor GM+PRP. The therapeutic effect of this agent was further investigated *in vivo* on a wound-healing model in rats. Wounds were locally injected with phosphate buffered saline (PBS), GM, PRP, and GM+PRP. Wound healing rate, vessel density, and inflammation level were measured histologically, by RT-PCR, and by Western blotting at days 3, 7, 14, and 21. Platelets on GM caused a continuous high release in both interleukin-10 and metalloproteinase-3 compared with PRP alone. Both GM+PRP and PRP successfully accelerated the wound healing process, while GM alone did not improve the wound healing process compared with the untreated control. Wounds treated with GM+PRP resulted in shorter healing period and improved dermal structure. GM+PRP improved angiogenesis in the wound by increasing expression of angiogenic factors. GM+PRP prolonged and enhanced the cytokine release profile compared with PRP. By promoting the inflammatory and angiogenic responses, GM+PRP has the potential to improve wound healing. Our findings demonstrate that GMs are an injectable carrier that enhanced the therapeutic effects of PRP.

## Introduction

Wound healing, including inflammation, proliferation and remodeling, is a dynamic process 1. Chronic diseases and aging can interrupt the wound healing process, even though patient morbidity and the risk of infection may be lowered due to early wound closure 2, 3. This disruption may cause chronic wound and hypertrophic scar formation, which can complicate treatment.

Advances in tissue engineering and regenerative medicine, like tissue-engineered skin substitutes, have brought numerous benefits 4. However, tissue-engineered skin substitutes have their own limitations. Culturing autologous skin substitute takes time, and transplanting allogeneic skin substitute runs the risk of immune rejection.

Platelet-rich plasma (PRP) belongs to the plasma component and has high concentration in rich platelet compared with baseline 5, 6. Chemokines, cytokines, matrix proteins, and growth factors are abundantly found in PRP 7-9. PRP is usually prepared spontaneously from blood plasma to heal chronic and acute wounds 10-13. Especially in the treatment of ulcerative diseases, the healing effect of PRP is more noticeable. Certain growth factors found in PRP like epidermal growth factor (EGF), platelet-derived growth factor (PDGF), transforming growth factor-β (TGF-β), and vascular endothelial growth factor (VEGF) bring about beneficial effects on wound healing 14-16. Therefore, research on PRP regeneration has attracted extensive attention in the fields of regenerative medicine and tissue engineering. At present, the application of PRP in clinical science involves many fields, such as orthopedics, dentistry and ophthalmology 17. The clinical effectiveness of non-activated autologous platelet-rich plasma (A-PRP) and activated autologous platelet-rich plasma (AA-PRP) for the treatment of Androgenic alopecia (AGA) and pattern hair loss had been demonstrated in recent reports 18-21, which had confirmed that A-PRP and AA-PRP had significant efficacy and no side effects 22, 23. In orthopedics, PRP is injected into patients with knee arthritis to relieve pain and promote the proliferation of human synovium and chondrocytes 24. The use of PRP after tooth extraction can accelerate wound and cartilage tissue healing. Treatment of dormant corneal ulcers, soft tissue defects, and hidradenitis suppurativa with PRP has shown positive outcomes 18, 25.

Despite the potential benefits of PRP in wound healing, currently it is limited by its low localized growth factors concentration, short half-life, and lack of standardized preparation methods 22, 26, 27. The growth factors in PRP need to be secreted in 10 min but most growth factors are secreted in 1 h; therefore, the growth factors are diluted and rapidly decomposed into the bloodstream after injection. The concentrations of secreted growth factors and the platelet amount are linearly proportional. To address the aforesaid concerns, a biocompatible material was used to a set up a controlled system 28. Due to the similarity in components and structure, gelatin is a good implantable material in the clinic 23, 26, 28. The use of gelatin as a carrier of PRP retains growth factors at the application site, thus accelerating angiogenesis and wound healing 29-32. Gelatin plays an important role in wound treatment 18 and preservation of PRP 33. Research on gelatin microspheres (GMs) involves many fields such as biology, medicine, and chemistry. Recently, growth factors have been carried in GMs to be delivered. Compared with other forms of carriers, the advantages of microspheres are that they can extend the h55alf-life of growth factors that they carry 34, and consequently improve the therapeutic effect *in vivo* 22, 35*.* GMs loading PRP has been applied for tissue regeneration, such as treatment in the osteoarthritis in rabbit 36, treatment for the degenerated intervertebral disc (IVD) model in rabbit 37, 38, bone regeneration in rabbit 39, 40, and treatment of ischaemic disorders in mouse 41.

The aim of this study is to use GMs as a carrier to improve the effects of PRP on the process of wound healing. We examined the growth factor released from the biomimetic bioreactor compound (GM+PRP), and measured cell proliferation. Then, we injected the novel compounds into a rat wound model. Finally, we assessed wound healing rate, angiogenesis, and the inflammation level with biochemical and histological analysis to investigate the efficacy in treating full thickness wound.

## Material and methods

### Preparation of GMs

To prepare GMs, 1-ethyl-3-dimethylaminopropyl carbodiimide hydrochloride (EDC) as used for crosslinking gelatin in an emulsion state 42. At first, gelatin was dissolved in deionized water to give 10 wt% gelatin solution, which was then used to dissolve type A gelatin with isoelectric point of 5 (Ward's Science, NY, USA) in a hot-water bath.

Next, paraffin (50 mL, containing span 80 [1%, TCI, OR, USA]) was added dropwise to the 10 mL solution. Magnetic stirring was used to emulsify the mixture for 20 min. The lactescent mixture was then put on an ice bath to quickly cool, then the gelatin was gently stirred in cold solution for washing. Subsequently, the aqueous solution of gelatin was added to cold acetone (50 mL, 4 °C), and was centrifuged (4 °C, 1000 rpm) to collect GMs. Then, the remaining paraffin on the surface was removed by washing the microspheres in chilled acetone for three times, and then in evaporated acetone in a baking box. To crosslink the GMs overnight, the microspheres (1 g) with EDC (50 mM, ProteoChem, IL, USA) and DW/acetone (1:4, v/v) were added into a 50 mL container and then the mixture was stirred by magnetic stirring on low speed. For freeze-drying, the microspheres were rinsed three times in DW. The release profiles of IL-10 and TIMP-3 in the GM+PRP, GM, and PRP groups were assessed by ELISA. Upon being in contact with the activator, the growth factors were abruptly and immediately secreted by PRP, and were rapidly dispersed into the surroundings.

### PRP preparation and adhesion

Adult rats (weighed about 425 ± 25 g, n=3) received pentobarbital (dose 65 mg/kg body weight) administration by intraperitoneal injection. The tubes were added with 10% sodium citrate (0.5 ml) as anticoagulant to deposit the blood specimen (5 mL) that was extracted from the abdominal aorta. Then the specimen was centrifuged (10 min, 2516 *g*. To precipitate the platelets, plasma was collected and centrifuged (1000 g for 10 min). Then, PRP was obtained by suspending the precipitated platelets in the platelet-poor supernatant. To immobilize the PRPs onto the GMs, we added PRP solution (0.5 ml) carefully to the GM+PRP (50 mg) mixture and then placed the mixture in a thermostat at 37 °C for 1 h. We rinsed off the unattached PRP from the microspheres using DW wash off. The GM+PRP and GMs were coated with gold, and were examined by scanning electron microscopy (SEM) (Hitachi S-3000N, Japan). The number of platelets were 612 × 10^4^/µL (SD: 156 × 10^4^/µL) in PRP, and about 34% of the platelets were immobilized onto the GMs by physical absorption.

### Enzyme-linked immunosorbent assay (ELISA)

We used enzyme-linked immunosorbent assay (ELISA) to quantify growth factors' release profiles in different groups with the metalloproteinase-3 (TIMP-3) and IL-10 ELISA Kits (R&D Systems, Minneapolis, MN). We tested specimens strictly based on the manufacturer's instructions. The GMs were incubated with PRP for 1h, then the mixture was washed off with phosphate-buffered saline (PBS) to remove any unattached platelets. In order to fully activate the platelets, 4% CaCl_2_ (CaCl_2_: PRP = 7:1) was added into the buffer. Then, we added PBS solution to the samples and incubated them at 37 °C. Next, we collected the supernatants. This protocol was used for the pure PRP group and GM+PRP group.

### Cell isolation and culture

The skin sample was sliced into 1-2 mm^2^ of small parts that are devoid of hair and fat, which was then digested to isolate the cells using trypsin- ethylenediaminetetraacetic acid (TE, Mediatech, Herndon, VA, USA) and collagenase type I (Worthington, Lakewood, NJ, USA). The medium, composed of Ham's Dulbecco's modified Eagle's medium (DMEM): F12: Epilife (1:1:2; Gibco, Invitrogen, Grand Island, NY, USA), was used to culture the collected cells, and was replenished by fetal bovine serum (5%, FBS, PAA Laboratories, Pasching, Austria). In addition, Epilife medium was used for culturing keratinocytes.

### Transwell invasion and migration assay

Millipore Transwell chambers (pore size: 8 μm) was used to perform Matrigel invasion assay. We combined 500 μL of medium containing 15% FBS with 100 μL and added the mixture into the lower chamber of a 12-well plate to induce cell migration 43. Cells were treated with PBS (control), GM (1 g/L GMs), PRP (1 g/L PRP), and GM+PRP (4 g/L GM+PRP). We added 2×10^4^ keratinocytes coated with growth-factor reduced Matrigel (Corning), and serum-free medium (500 μL) into the upper chamber of the plate. The plate was incubated for 24 h at 37^◦^C. The upper surface of the membrane of cells was cleaned up using a cotton swab before incubation was over. After migration, the cells were treated with methanol (Merck) through the lower surface of the membrane and stained with Giemsa (HiMedia). The images of cells were obtained using an inverted microscope (CKX41, Olympus; Shinjuku, Tokyo, Japan). NIH ImageJ software (dVersion 1.46, Bethesda, MA, USA) was used to count the cells in five fields-of-view. The experiment was conducted in triplicate.

### Measurement for cell proliferation

The cytotoxicity of materials to keratinocytes was tested by Dojindo Cell Count Kit-8 (CCK-8; Kumamoto Techno Research Park, Tokyo, Japan). Cells were implanted in 96-well plates with 3×10^3^ cells for each well; treated with GM (1 g/L GMs), PRP (1 g/L PRP), and GM+PRP (4 g/L GM+PRP) 43; and incubated under different lengths of time after adhesion (1, 3, 6, 12, 24 and 48 h). After replacing the culture media (100 μL DMEM), the plates were incubated for 2 h, and then 10 μL of CCK-8 was added. To calculate the relative growth rate (RGR), a microplate reader (Bio-Rad, Hercules, CA, USA) was used to examine optical density (OD) from the absorbance at 450 nm. The data were input into the computer software for calculation based on RGR = testing OD/controlled OD. The blank group of ASCs plus PBS was used as the standard, and the cell viability calculated by other groups was compared.

### Wound healing model

A rat excisional wound healing model was used to assess the ability of the stromal GM+PRP for enhancing wound healing. We set a light-dark cycle of 12 h, and provided food and water to the rats (female Sprague-Dawley, 6-8 weeks old, n = 40), which were individually treated. Articaine (0.2 mg/kg body weight, hypodermic injection) was used for subdermal anesthesia before the operation. Punch biopsy was used to create wound of diameter 3 mm on the skin on the back of the rats. Body temperature was maintained throughout the operation using a heating cushion. Erythromycin eye ointment was administrated to protect the cornea from drying. The 40 rats were divided equally into four groups: Control, GM, PRP, and GM+PRP. As a control, 0.2 mL PBS was injected into the rats. The GM group was given 10 mg of GM suspended in PBS (0.2 mL). The PRP group was treated with PRP injection (0.2 mL). The GM+PRP group was treated with 10 mg GM+PRP suspended in PBS (0.2 mL). All injections were performed subdermally into the four different quadrants of the wound. Until the wound heals or reaches day 21, the wound was wrapped daily by Tegaderm sterile dressing (3M Healthcare, St Paul, Minn.) without interruption. The photos were taken at 4 different time points during, and at 3, 7, 14, 21 days after the operation. ImageJ software was used to quantify wound proportions.

### Histological and immunohistological assessment

Hematoxylin & Eosin (HE Stain) was used for histological analysis and each harvested specimen was analyzed at 3, 7, 14 and 21 days. Masson staining was also used to evaluate the density of collagen in the wounded skin according to the manufacturer's protocol. Hoechst 33342 was used to stain the nuclei (dilution 1:500, Thermo Fisher Scientific, ON, Canada). Immunohistochemistry was carried out on day 21 after modeling. The main antibody evaluation was implemented through mouse monoclonal F4/80 (dilution 1:200, Abcam, ON, Canada), and 3, 3'-diaminobenzidine (DAB) was used to visualize the target protein. Images were captured using Nikon 90i microscope.

### Western blot

Skin tissues were lysed in radioimmunoprecipitation assay (RIPA) buffer on ice for 30 min. We measured protein expression from the cell lysate (30~60 mg) using IL-6 (ab6672, Abcam, MA, USA), IL-10 (12163, CST, MA, USA), VEGF (ab32152, Abcam, MA, USA), MCP-1 (ab25124, Abcam, MA, USA), and β-actin (3700, CST, MA, USA). Briefly, the samples were loaded onto 15% SDS gel to separate the proteins by polyacrylamide gel electrophoresis. Then the proteins were transferred onto the PDVF membrane (ISEQ00010, Millipore, MA, USA). Primary antibodies were incubated for 12 h at 4 °C. Washed membrane and then incubated it with the HRP-conjugated antibody, and finally conducted with ECL. The gray value of the bands was analyzed using a gel analyzer (Media Cybernetics, MD, USA).

### Statistical analysis

Data were analyzed by one-way ANOVA with Bonferroni post-hoc power analysis. For the wound healing analysis, data were analyzed by a two-way ANOVA with Bonferroni post-hoc analysis. Statistical significance between two or more groups was determined by Bonferroni/Dunn's test. Statistical analysis was conducted by Graphpad Prism 7.0 (Graphpad Software Inc., San Diego, CA, USA), and all data shall be expressed in the form of mean ± standard error. Significance level was set at *P* < 0.05.

## Results

### Morphology of GMs and GM+PRP

**Figure 1:**
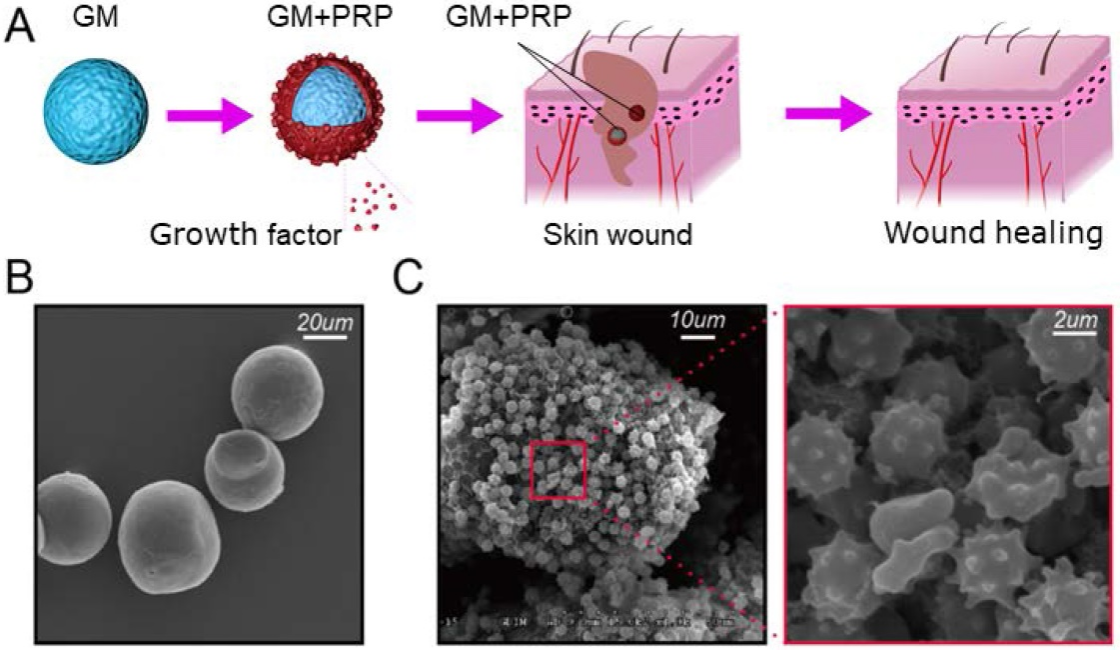
Schematic illustration. (A) GMs loading with PRP. (B) SEM on GMs with smooth spherical surface. (C) Immobilized PRP on GMs.

Schematic illustration of GMs loaded with PRP is presented in Figure 1A. As shown by SEM images, numerous platelets were stabilized on the GM surface and some platelets were activated (Figure 1B, C). The diameter of the GM was 32.02±5.01 µm.

### GM+PRP enhanced and prolonged release profile

**Figure 2:**
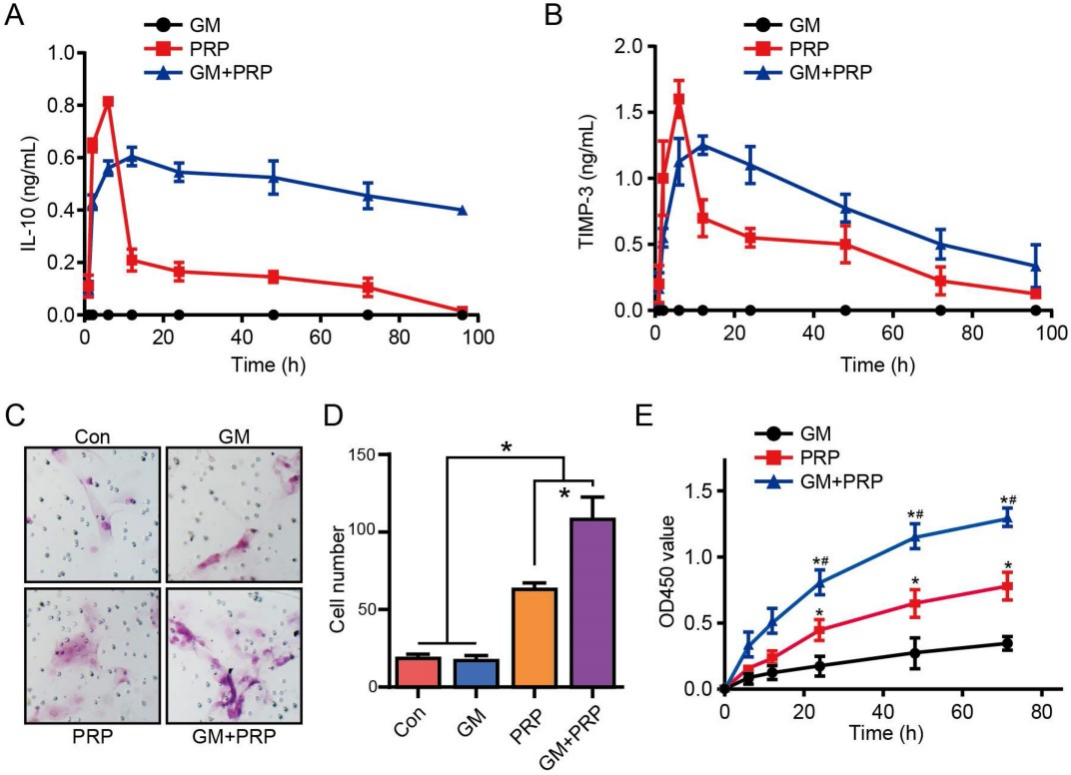
Effect of GM+PRP on release profile migratory properties of keratinocytes. (A) Release profile for IL-10. (B) Release profile for TIMP-3. (C) Transwell invasion migration assay. (D) Cell number. *P < 0.05. (E) The CCK8 assay used to evaluate the proliferation of keratinocytes after incubating with GM, PRP and GM+PRP. One-way ANOVA by LSD post hoc. *P < 0.05 compared with the GM group, #*P* < 0.05 compared with the PRP group.

The GM+PRP group had prolonged and higher release profile compared with the PRP group. IL-10 level in the GM+PRP group detectable for up to 100 h but it was undetectable in the PRP group after 100 h. After 24 h, IL-10 level in GM+PRP group was markedly higher than that in PRP group (*P <0.05) (Figure 2A). TIMP-3 levels followed a similar trend as the IL-10 levels. After 24 h, TIMP-3 level was markedly higher in GM+PRP group than that in PRP group (*P <0.05) (Figure 2B).

### GM+PRP improved migratory properties and proliferation ability of keratinocytes

Migratory properties of keratinocytes play an essential role in wound healing. We compared the four groups to investigate the effect of PRP on keratinocyte migration (Figure 2C). There was a significant increase in the migration of keratinocytes after PRP and GM+PRP treatment (*P < 0.05, respectively). There was a statistically significant (*P < 0.05) difference between GM+PRP group and PRP group, while the comparison between control group and GM group showed no significant (*P* > 0.05) difference (Figure 2D). CCK8 proliferation assay showed that PRP increased cell proliferation more than the GM group did, and cell proliferation in the GM+PRP group was higher than that in the PRP group (Figure 2E).

### GM+PRP promoted wound healing

**Figure 3:**
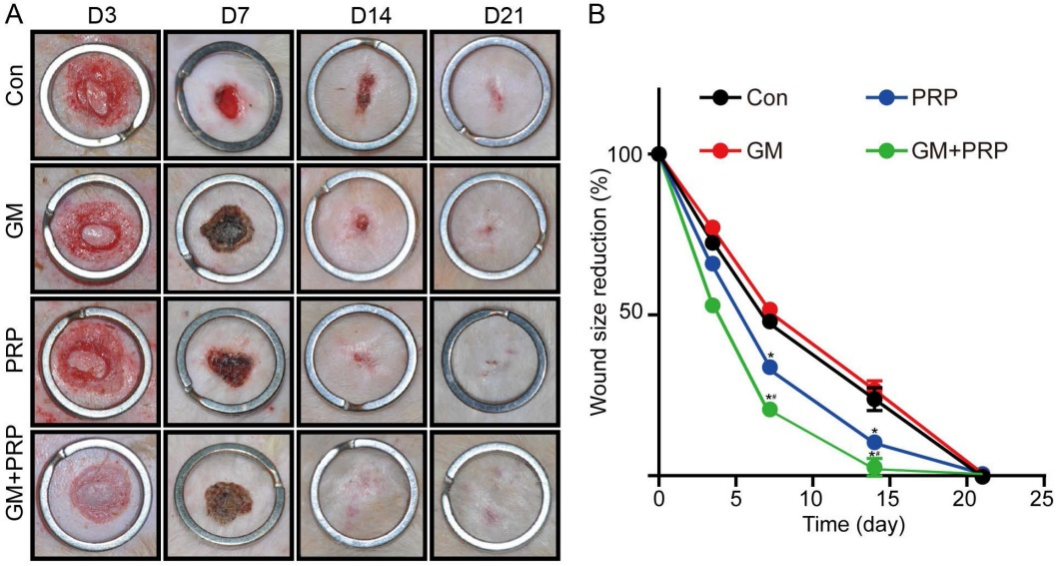
Treatment influences of GM+PRP, PRP and GM in wound healing. (A) Representative wound pictures on day 3, 7, 14, and 21 after surgery. (B) Percentage of wound area observable each day. *P < 0.05 compared to the control (Con) group, #*P* < 0.05 compared to the PRP group at the same time point.

After wound creation, each wound randomly received one of the four treatments: GM+PRP, PRP, GM or PBS. Compared with the control group (*P* > 0.05) and the group treated with the stromal vascular fraction cell suspension, there were no apparent improvement in wound size in the treated group with GM+PRP in the early phase of repair (0-3 days). However, the wound size in the GM+PRP group was smaller than that in the other groups (*P < 0.05) from day 3 to 14. Also, a complete wound healing was observed in the GM+PRP group at day 14. In contrast the other groups did not show complete wound closure at day 14 (Figure 3).

### GM+PRP increased collagen density and enhanced angiogenesis

**Figure 4:**
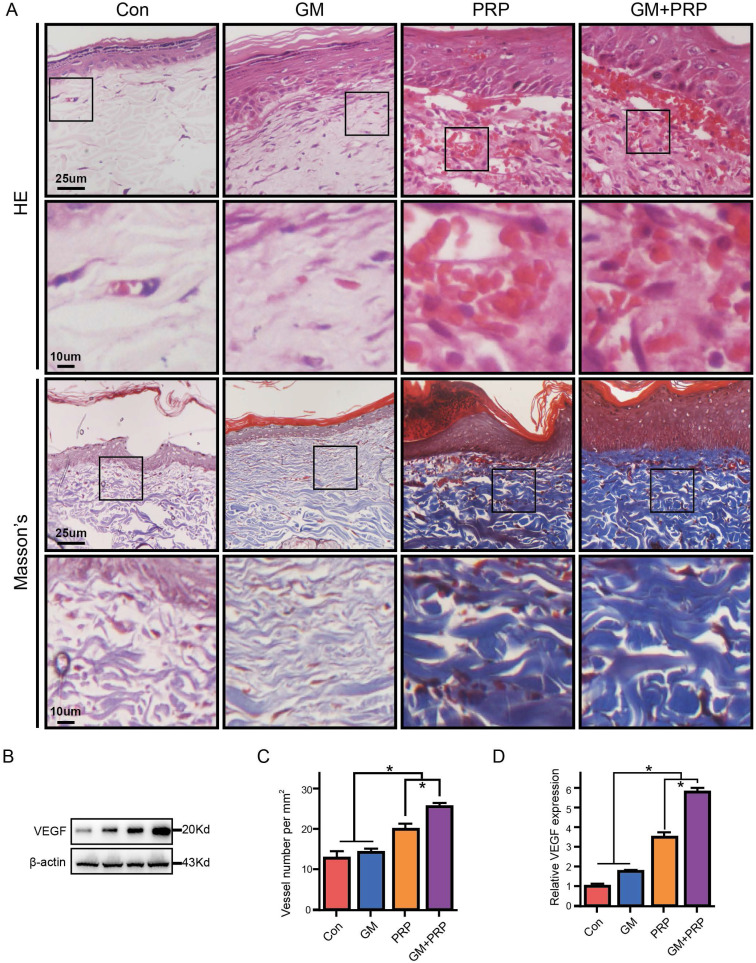
Effect of GM+PRP on collagen density and enhanced angiogenesis. (A) Hematoxylin & eosin staining of wounds on day 21 (upper) and Masson staining of wounds on day 21 (below). (B) Western-blot analysis of VEGF in wounds. (C) Quantification of blood vessels in wounds. (D) Statistic analysis of VEGF protein in each group wounds. (E) qRT-PCR analysis of VEGF in wounds. *P < 0.05.

Re-epithelialization in all the wounds was observed on day 21 based on histological analysis. Particularly, skin appendage regeneration, and tight connection between dermis and epidermis were observed in the GM+PRP group. However, skin appendage regeneration and loose connection between the dermis and epidermis were found in other groups (Figure 4A). Collagen density of the wounded skin was evaluated by Masson's trichrome staining and the results showed that both GM+PRP and PRP increased collagen density in the dermal layer of the healing wound, while the GM group and the control group shared no difference in collagen density (Figure 4A). Blood vessels were counted in HE stain, and the results showed that tube-structure blood vessels in the GM+PRP group were higher than those in the other groups (Figure 4C). Protein level (Figure 4B) and gene level of VEGF expression (Figure 4D, E) in the wound were evaluated; the results showed that GM+PRP treatment significantly increased the protein level and gene expression of VEGF.

### GM+PRP increased local inflammation in the wound

**Figure 5:**
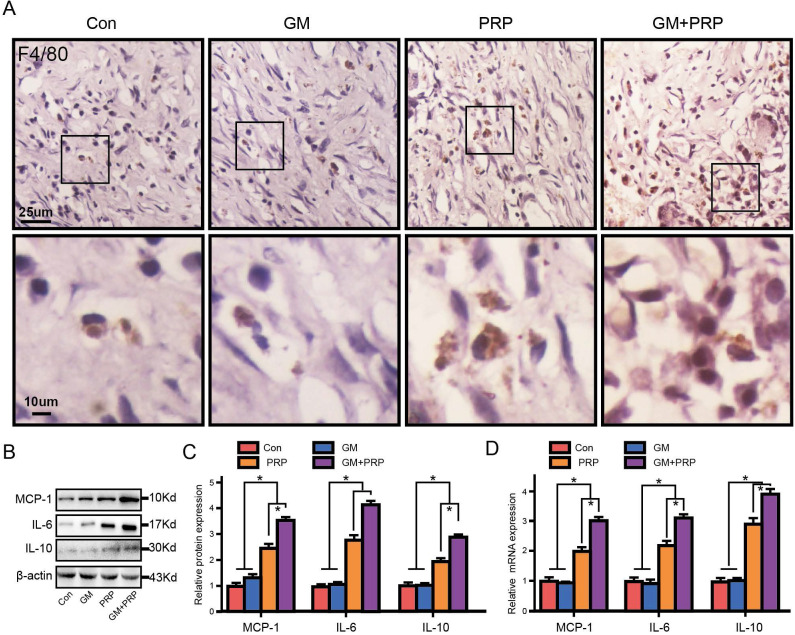
Effect of GM+PRP on local inflammation in the wound. (A) Immunochemistry of F4/80 in wounds. (B) Western-blot analysis of inflammation-related cytokines expression in wounds. (C) Band density. (D) qRT-PCR analysis. *P < 0.05.

Immunochemistry of F4/80 showed that both GM+PRP and PRP increased local macrophage infiltration in the dermal layer (Figure 5A). The expression of the inflammatory cytokines IL-6, IL-10, and MCP-1 by Western blot is shown in Figure 5B. On day 7, protein level of MCP-1 was markedly increased in the groups of GM+PRP and PRP compared to the control group (Figure 5C). Moreover, the GM+PRP group had significantly higher-level expression of IL-6 compared to that of the PRP group (Figure 5C). On day 7, GM+PRP significantly increased IL-10 expression in GM+PRP group (Figure 5C). Similar trend was also demonstrated by the Western blot test (Figure 5D).

## Discussion

It remains a challenge to develop new efficient approaches for therapy based on the wound healing mechanism and regenerative medicine. Due to diabetes and other conditions, it is necessary to resolve chronic wounds with new effective therapies. Topical application of growth factors that promote wound healing is one potential alternative. PRP - an autologous agent enriched with high concentration of platelets and various growth factors - is regarded as a promising therapeutic option to enhance the wound healing process 44. The earlier preparation of PRP can go back to the 1970s 45. However, reliable ways to ensure the sustained release of growth factors and enhance the long-term therapeutic effect are required for further clinical application of PRP 46, 47. In this study, GMs were loaded with PRP to fabricate biomimetic bioreactor gelatin microspheres (GM+PRP). We then measured its efficacy in treating full thickness wound. GM+PRP ensured prolonged growth factor VEGF release and improved full thickness wound in rats. Platelets and functional proteins from the PRP were immobilized onto GMs. Our findings suggest that GM+PRP enhances the wound healing rate and reduce the wound healing time by increasing macrophage infiltration and inflammatory cytokines. Our results indicate that GMs is an injectable carrier that enhanced the therapeutic effect of PRP.

Our study confirmed that the mRNA and protein expression levels of VEGF in the wound were up-regulated (p<0.05), indicating that GM+PRP induced the transcription and expression of endogenous VEGF. GM+PRP significantly increased the area containing new capillaries and promoted the formation of microvascular network in the subcutaneous tissue (Figure 4). Another study has shown that PRP with gelatin hydrogel granules significantly enlarged the area containing newly formed capillaries, and promoted the microvascular network in murine subcutaneous tissue 41, thus the full-thickness wound in rats was improved. The PRP components contain VEGF, IL-10, TIMP-3, TGF-β1, PDGF, insulin-like growth factor (IGF)-1, and stromal-cell derived factors (SDF-1) 5, 6, 13 .These proteins are key in wound healing, as shown by the beneficial role of exogenous growth factors in wound healing, and *in vitro* activity of many growth factors and cytokines 2, 48. One study presented that PRP in association with insulin regulated Akt mechanism and mTOR signalling 9, 24. Platelets are actively involved in biological processes such as tissue regeneration and inflammation 49-51. The phenomena are mediated by the storage of alpha-granule particles that release platelets including extracellular matrix modulators, growth factors, and cytokines 23, 49. Those cytokines and secretory proteins could contribute to the healing process by different mechanisms. In clinic, the effect of gelatin sheet combined with PRP has been reported 33; the surface area of GMs increased, thus, more PRP could be immobilized onto the GMs. Furthermore, the sheet could sustain the release of factors, and the sheet impregnated with factors promoted wound closure 36. In this study, as the surface area of GM increases, more PRP could be immobilized onto the GMs, stimulating neo-epithelialization and neovascularization in murine skin defects. The type A gelatin has a positive charge at physiological pH due to an isoelectric point of 5 29, 33. Once a basic growth factor was ionically immobilized in the acidic gelatin hydrogel, which can be controlled by biodegradation of the biodegraded hydrogel 52. Another study showed that the isoelectric point of GMs affected the release rate of growth factors adsorbed on the microspheres, and the degradation rate of GMs played a decisive role in the release rate of growth factors adsorbed on the microspheres 34. Thus, more PRP could be immobilized onto the GMs, which ensured prolonged release of VEGF thereby up-regulating the mRNA and protein expression levels.

In this study, compared with PRP alone, GM+PRP prolonged and enhanced cytokine release profile, and showed greater healing potential in acute wound. If there is no stabilizer, PRP will immediately secrete growth factors after exposure to the activator, and the released growth factors will rapidly disseminate in the surrounding environment, which compromises its long-term effect 53-55. The release profile in GM+PRP group was more prolonged and higher compared with the PRP group (Figure 2). Platelets on the microspheres' surface released a portion of the growth factors, which were loaded by a carrier of gelatin through a physical interfacial interaction. From SEM results, it is observed that platelets attached to the microspheres' surface layer by layer 56. The crowded platelet in a layer-by-layer structure hindered the activation of platelets, thus, prolonging the activation of platelets and causing higher and prolonged release of functional proteins from the platelets. It is found that hydrogel with two/three-dimensional structure is a good carrier to deliver the PRP to wound sites, hydrogel works as a scaffold or vehicle, and PRP can loaded in hydrogel as the two main forms, e.g., PRP gel 11, 57, 58 and hydrogel microsphere 36-38. PRP can be incorporated in hydrogel (like gelatin, chitosan, collagen and alginate) as a gel, which is a semi-solid state. While the introduction of hydrogel microspheres such as GMs have been given in the above descriptions of this study. Compared with gels, hydrogel microspheres like GMs have better mechanical properties, and can provide a 3D micro-environment for cells growth 59.

In this study, we found an immune-regulatory function of PRP that improved wound healing process (Figure 4). Both PRP and GM+PRP increased macrophage infiltration and upregulated inflammatory cytokines, and ultimately contributed to accelerating wound healing 60. The recovery of damaged connective tissues can be improved by activation, migration, and proliferation of fibroblasts. The synergetic action of these factors - by inducing migration, differentiation, proliferation, and stabilization of endothelial cells in new blood vessels - sequentially regulate inflammation levels and enhance revascularization of damaged tissues. Moreover, it can increase stem cell recruitment, differentiation, and proliferation to tissue-specific cell types 61.

It is crucial to eliminate devitalized tissues and cellular debris and to avoid infection through immune and inflammatory pathways. Wound healing is a physiological and dynamic process that involves sequential stages of acute inflammation, formation and proliferation of new tissues, and remodeling to restore the regular structure and function of the damaged tissues 6. After tissue damage, wound repair instantly takes place to form platelet embolism, and then a fibrin matrix is consolidated to turn into a scaffold for cell attachment. Monocytes differentiate into M1 macrophage phenotype after recruitments of monocytes and neutrophils 62.

In the current study, both GM+PRP and PRP increased macrophage infiltration on day 7. MCP-1 has a vital function in the site of inflammation to recruit mononuclear cells 63 and was significantly enhanced in the GM+PRP and PRP groups 42, 64. MCP-1 expression could be transiently induced by platelet-derived products, such as serotonin, PDGF-BB, or PDGF-AB 65. Moreover, pro-inflammatory cytokines (e.g., IL-6) were also significantly increased in the GM+PRP group, indicating that inflammatory cells were highly active during this period to remove cellular debris and stimulate angiogenesis, providing a favorable environment for tissue regeneration.

Mesenchymal cells, endothelial cells, granulocytes, fibroblasts, and macrophages can synthesize and release growth factors after obtaining a stimulus from platelets 66. Anti-inflammatory cytokines were significantly upregulated in the GM+PRP group. High levels of anti-inflammatory cytokines play an important role in transforming pro-inflammatory phase and transitioning to regeneration phase. The GM+PRP group had both active pro-inflammatory and anti-inflammatory phases. Due to the dysregulation, complex diseases may be exacerbated and may even prevent repair; thus, rapid pre-inflammatory phase and transition to regeneration phase are critical to damaged tissues 48, 67, 68. However, as with many tissue-engineered skin substitutes, the risk of immune rejection between gelatin and biological tissues is unavoidable, and the process of injecting PRP into GMs is extremely time-consuming.

## Conclusion

GM+PRP prolonged and enhanced cytokine release profile compared with PRP, thus showing greater healing potential in acute wound. GM+PRP may have the potential to accelerate the wound healing process by increasing macrophage infiltration and upregulating inflammatory cytokines. These findings indicate that an injectable carrier, GM, enhanced the therapeutic effect of PRP.
